# Updates on Molecular and Biochemical Development and Progression of Prostate Cancer

**DOI:** 10.3390/jcm10215127

**Published:** 2021-10-31

**Authors:** Omar Fahmy, Nabil A. Alhakamy, Waleed Y. Rizg, Alaa Bagalagel, Abdulmohsin J. Alamoudi, Hibah M. Aldawsari, Aiah M. Khateb, Basmah M. Eldakhakhny, Usama A. Fahmy, Wesam H. Abdulaal, Claudia G. Fresta, Giuseppe Caruso

**Affiliations:** 1 Department of Urology, Universiti Putra Malaysia, Serdang 43400, Malaysia; docomar82@gmail.com; 2 Department of Pharmaceutics, Faculty of Pharmacy, King Abdulaziz University, Jeddah 21589, Saudi Arabia; nalhakamy@kau.edu.sa (N.A.A.); wrizq@kau.edu.sa (W.Y.R.); haldosari@kau.edu.sa (H.M.A.); uahmedkauedu.sa@kau.edu.sa (U.A.F.); 3 Advanced Drug Delivery Research Group, Faculty of Pharmacy, King Abdulaziz University, Jeddah 21589, Saudi Arabia; 4 Center of Excellence for Drug Research and Pharmaceutical Industries, King Abdulaziz University, Jeddah 21589, Saudi Arabia; 5 Mohamed Saeed Tamer Chair for Pharmaceutical Industries, King Abdulaziz University, Jeddah 21589, Saudi Arabia; 6 Department of Pharmacy Practice, Faculty of Pharmacy, King Abdulaziz University, Jeddah 21589, Saudi Arabia; abagalagel@kau.edu.sa; 7 Department of Pharmacology and Toxicology, Faculty of Pharmacy, King Abdulaziz University, Jeddah 21589, Saudi Arabia; ajmalamoudi@kau.edu.sa; 8 Medical Laboratory Technology Department, College of Applied Medical Sciences, Taibah University, Madinah 42224, Saudi Arabia; akhateb@taibahu.edu.sa; 9 Department of Clinical Biochemistry, Faculty of Medicine, King Abdulaziz University, Jeddah 21555, Saudi Arabia; beldakhakhny@kau.edu.sa; 10 Department of Biochemistry, Faculty of Science, Cancer and Mutagenesis Unit, King Fahd Medical Research Center, King Abdulaziz University, Jeddah 21555, Saudi Arabia; whabdulaal@kau.edu.sa; 11 Centre for Artificial Intelligence in Precision Medicines, King Abdulaziz University, Jeddah 21555, Saudi Arabia; 12 Department of Biomedical and Biotechnological Sciences, University of Catania, 95125 Catania, Italy; forclaudiafresta@gmail.com; 13 Department of Drug and Health Sciences, University of Catania, 95125 Catania, Italy

**Keywords:** prostate cancer progression and tumorigenesis, biomarkers, growth factors, inflammation, oxidative stress, androgen deprivation therapy

## Abstract

Prostate cancer (PCa) represents the most commonly non-cutaneous diagnosed cancer in men worldwide and occupies a very wide area of preclinical and clinical research. Targeted therapy for any cancer depends on the understanding of the molecular bases and natural behaviour of the diseases. Despite the well-known effect of androgen deprivation on PCa, many patients develop resistance either for antiandrogen therapy or other new treatment modalities such as checkpoint inhibitors and chemotherapy. Comprehensive understanding of the development of PCa as well as of the mechanisms underlying its progression is mandatory to maximise the benefit of the current approved medications or to guide the future research for targeted therapy of PCa. The aim of this review was to provide updates on the most recent mechanisms regarding the development and the progression of PCa. According to the current understanding, future treatment strategies should include more predictive genetic and biomarker analysis to assign different patients to the expected most appropriate and effective treatment.

## 1. Introduction

Prostate cancer (PCa) represents the most common cancer among men after cutaneous melanoma, occupying the second place with regard to male cancer mortality worldwide [1]. In United States, more than 170,000 new cases are diagnosed every year due to PCa, while more than 31,000 people die because of this aggressive type of cancer [2]. In the near future, the number of new cases is expected to rise, especially in accordance with the fact that life expectancy is globally increasing [3]. One of the main challenges in the management of PCa is the unexpected behaviour of the disease in some patients. Patients with low International Society of Urological Pathology (ISUP) grade are usually responsive to treatment, while others with high ISUP grade display progression and metastasis with poor prognosis [4].

In view of the well-known androgen sensitivity of PCa, patients with metastatic or recurrent disease, despite the treatment, are usually subjected to androgen deprivation therapy (ADT), consisting of luteinizing hormone releasing hormone (LHRH) agonists or LHRH antagonists [5]. Despite the effective suppression of androgen signals, many PCa patients will eventually transform into castration-resistant PCa (CRPC), which is characterised by a high rate of metastatic disease (mCRPC) and a poor prognosis. Eventually, it causes symptoms and, in the worst case scenario, death among PCa patients [6].

Recently, both chemotherapy and immunotherapy have been given an evolving role in the management of PCa. Indeed, docetaxel chemotherapy administered to patients with mCRPC has represented the standard therapy since 2004, with a minimal survival benefit [7]. Yet, recent data from two landmark randomised studies (CHAARTED and STAMPEDE) displayed that combination of docetaxel and ADT in patients without previous ADT resulted in more than one year overall survival benefit compared to ADT alone [8,9]. Immunotherapy was introduced among several options for men with mCRPC in earlier times; however, the clinical benefit of immunotherapy remains inconclusive in unselected patients. In the new era of immune checkpoint inhibitors (ICIs), these new medications such as programmed cell death protein 1 (PD-1)/programmed death-ligand 1 (PD-L1), and cytotoxic T-lymphocyte-associated protein 4 (CTLA-4) inhibitors have been showing promising results through the stimulation of anti-tumoral immunity. In fact, there is a growing body of evidence that shows how the use of ICIs could be more beneficial in PCa patients belonging to a specific sub-group characterised by high PD-L1 tumour expression or high tumour mutational burden [10].

There is currently a dearth of clarity regarding the cellular pathways and molecular underpinnings of PCa progression. Shedding more light on the molecular pathways driving the genesis and progression of PCa is critical for the identification of potential therapy targets as well as to decrease the mortality of this disease.

## 2. Histological Background of the Prostate Gland

The prostate gland has three primary glandular areas that are not identical in terms of histological and molecular aspects: the peripheral zone, the core zone, and the transition zone [11]. The transition zone is the primary location of formation of prostate hyperplasia, while the core zone is relatively resistant to cancer and other disorders. Several significant non-glandular regions are also localised in the anteromedial section of the gland [12]. Secretory epithelium lines both ducts and acini in all zones. There is a layer of basal cells underlying the secretory lining in each zone. Their presence differentiates between benign and malignant glands [13].

The gland has a stroma that is made up with connective tissue and smooth muscle fibres. The prostatic stromal contains several components that are anatomically and physiologically important for the gland’s proper function. Changes in several of these stromal variables could contribute to the development and progression of PCa. Indeed, prostate tumour development and metastasis are dependent on the interaction of neoplastic cells with stromal components [14]. Fibroblasts play an important role in the prostatic stroma. These cells maintain epithelial cells intact by continually modifying and interacting with diverse components inside the organ [15]. Fibroblasts contribute to the production of extracellular matrix through the secretion of collagen types I and III, and are also important for tissue healing by regulating the formation of granulation tissue and the transition into myofibroblasts. In the case of prostatic neoplastic transformation, stromal smooth muscle cells are replaced by cancer-associated fibroblasts (CAFs), which are specialised fibroblasts. Cancer stroma is also responsible for the increase of the expression of fibroblast-specific markers such as vimentine, fibroblast specific proteins (FSPs), and alpha-smooth muscle actin (α-SMA), while the expression of demine is decreased [16]. CAFs represent the main source of angiogenesis and alteration factors of extracellular matrix components, including transforming growth factor beta (TGF-β), interleukin-6 (IL-6), growth differentiation factor 15 (GDF15), and fibroblast growth factor (FGF) [17]. The hypothesised activity of CAFs due to its contact with tumour cells leads to the development of an unregulated “reactive stroma,” which stimulates cancer cell proliferation and aggressiveness, also influencing treatment response [18].

## 3. Growth Factors Involved in Prostate Cancer

### 3.1. Insulin-like Growth Factor (IGF)

Different in vitro and in vivo preclinical studies have highlighted the role played by IGF system in the development of PCa, but despite the promising data, most of the clinical studies failed to demonstrate a direct link between the activity of the members of IGF family and the progression of PCa [19]. However, IGFs could play a role in certain group of patients depending on several factors. For instance, a recent comprehensive systematic review and meta-analysis suggested a link between milk ingestion and PCa through the modulation of IGFs [20]. Another clinical study linked the prognosis of PCa to the overexpression of IGFR-1 receptor in transmembrane serine protease 2-erythroblast transformation-specific-related gene (TMPRSS2-ERG) (T2E) gene-negative subgroup of patients [21].

### 3.2. Vascular Endothelial Growth Factor (VEGF)

This factor plays a key role in PCa growth by stimulating angiogenesis and formation of new vascularisation [22]. There are many receptors involved in the regulation of VEGF pathway; however, VEGFR1 and VEGFR2 are the main receptors involved in PCa. These two subtypes of receptors are more expressed in PCa compared to benign prostatic hyperplasia (BPH) [23]. In malignant conditions, and due to the rapid growth, malignant cells could be compressed by surrounding cells and this can induce hypoxia, that in turn leads to VEGF upregulation through the release of hypoxia-inducible factor 1 (HIF-1) [24]. Despite the potential role for VEGF in PCa, clinical trials considering the use of VEGF inhibitors did not show clinical benefit for PCa patients [22].

### 3.3. Platelet-Derived Growth Factor (PDGF)

PDGF represents a potent mitogen for the proliferation of fibroblasts and smooth muscle cells, both types of cells part of the prostate stroma. It could also play a role in the angiogenesis process [25]. Since PDGF receptor α (PDGFRα) has been detected in a significant amount in bone metastasis due to PCa, a role for the expression of this receptor in the progression of PCa as well as skeletal metastasis has been proposed [26]. Experimental preclinical studies reported the inhibition of PCa growth and progression in mice following the administration of imatinib, a tyrosine kinase inhibitor, in combination with paclitaxel [27]. In contrast, clinical studies revealed no clinical benefits, or even acceleration of disease progression. These controversial results lead to the hypothesis that PDGF can play the role of homeostatic factor in bone metastases and that the regulation of pericytes’ activity by PDGFRα could represent a gatekeeper for metastases [28].

### 3.4. Fibroblast Growth Factor (FGF)

FGFs represent a group of cell proteins produced by macrophages involved in the physiological development of cells. Any abnormality in their function can be the cause of aberrant growth or tumorigenesis [29]. There are two types of FGFs: paracrine and endocrine. Paracrine FGFs act as growth factors by activating the tyrosine kinase pathway through direct binding to the extracellular FGF receptors. Meanwhile, the endocrine FGFs circulate in the serum forming complexes with co-receptors, finally binding to the extracellular FGF receptors [30]. It has been shown by using PCa cell lines that FGF receptors display a heterogeneous pattern of expression. For instance, fibroblast growth factor receptor 2 IIIb (FGFR2IIIb) was detectable in LNCap cells that displayed androgen-dependent growth paralleled by a relatively low potential of cell proliferation. In contrast, this receptor was undetectable in PC3 cells that displayed androgen-independent growth and high potential of cell proliferation [31]. Some clinical trials employing FGF inhibitors have shown promising results, as it has been seen for the treatment of mCRPC with dovitinib and nintedanib [32].

### 3.5. Transforming Growth Factor β (TGF-β)

TGF-β is a multifunctional factor with three different receptors (types I, II, and III) directly involved in the modulation of its activity [33,34]. This grown factor plays a role in the angiogenesis process through the stimulation of both VEGF and connective-tissue growth factors (CTGF) in epithelial cells and fibroblasts [35]. Poor prognosis and higher grade of PCa has been noticed in patients with decreased or missing expression of TGF-β receptors types I and II; however, the value of this observation is limited by the low number of patients involved in the study [36].

### 3.6. Epidermal Growth Factor (EGF)

The ability of EGF to enhance cellular growth is mediated by the interaction with its receptor EGFR [37]. The activation of EGF signalling could be involved in PCa metastasis and progression through the suppression of ETS variant transcription factor 6 (ETV6), a tumour suppressor gene [38]. A recent study by employing a xenograft model showed that the disruption of ETV6 leads to TWIST1-dependent progression and resistance to EGFR tyrosine kinase inhibitors in PCa; on the basis of the results of this study, the authors proposed that ETV6 might represent a possible marker for predicting the efficacy of an EGFR-targeted therapy [39].

## 4. Effect of Inflammatory Modulators on PCa

Chronic inflammation is the physiological response of the tissue after the exposure to various forms of tissue injury [40]. This inflammatory reaction results in a sequence of chemical reactions and release of cytokines targeting the elimination of the causative factors and the restoration of the normal tissue architecture [41]. However, when the noxious element persists and/or the tissue is repeatedly exposed to it, the inflammatory reaction persists, moving from an acute to a chronic response; by doing so, permanent damage and alteration of the microenvironment occurs, leading to an uncontrolled proliferation of cells and enhanced genomic instability [42]. Cytokines are able to stimulate the proliferation of malignant cells as well as to affect the apoptotic process. Furthermore, they can enhance cancer cell migration causing metastasis [43]. Chronic inflammation represents a well-known risk factor for certain solid organ malignancies due to the related DNA damage. It has been shown that men with chronic prostatitis could have a higher risk of developing high grade PCa, even though it can be considered an association rather than a causative relationship [44].

There are many factors, either internal or external, that can initiate the inflammatory process in the prostate, as in the case of *E. coli*, which can reach the prostate through intraprostatic reflux of urine, or bacteria responsible for sexually transmitted diseases such as Neisseria gonorrhoeae. Dietary elements and lifestyle-risk factors are potential initiators of prostatic inflammation [45].

In PCa, chronic inflammation directly correlates with higher detection of proliferative inflammatory atrophy (PIA) lesions [46]. The PIA lesions observed at prostate level are often associated with increased acute or chronic inflammatory cell infiltration. Some of these lesions are characterised by an increased number of epithelial cells, while inflammatory cells are missing. Different phenotyping studies revealed an association between the existence of these lesions and prostatic intraepithelial neoplasia (PIN) and prostatic adenocarcinoma [47,48]. Surprisingly, these lesions are usually present in the peripheral zone of the prostate, which is the main site of PCa initiation [44].

Anti-inflammatory medications or ingestion of natural gradients with anti-inflammatory properties have been associated with the reduction of PCa risk. In REDUCE trial, aspirin and non-steroidal anti-inflammatory drugs (NSAIDs) were associated with lower risk of PCa in men with negative prostate biopsy [49]. The use of statins, able to inhibit 3-hydroxy-3-methyl-glutaryl-coenzyme A (HMGCoA), has been linked to a lower risk of advanced and aggressive PCa [50]. Two additional natural molecules characterised by anti-inflammatory activity, soy and green tea [51], have been associated with a reduced risk of PCa, possible due to their content of anti-inflammatory compounds such as genistein and daidzein [52].

## 5. Oxidative Stress in PCa

Oxidative stress has been defined as the imbalance occurring between the production reactive oxygen species (ROS) and cell antioxidant defences [53]. A plethora of publications has shown that the increased production of ROS and reactive nitrogen species (RNS) is linked to aging processes and to the etiopathogenesis of aging-related diseases, such as Alzheimer’s disease and cancer [53,54,55]. In particular, oxidative stress has been associated with PCa development and progression as well as to the response to the therapy. Oxidative stress has also been identified as one of the factors negatively modulating the development of an aggressive phenotype. In PCa, the most abundantly reported reactive species produced are represented by superoxide, hydroxyl radical, and nitric oxide (NO) [56,57]. It has also been observed an increased production of peroxynitrite, representing a very reactive and toxic reaction product of superoxide and NO [58]. The reduced expression of glutathione-S-transferase P1 (GSTP1) and nuclear factor-erythroid 2 p45-related factor 2 (Nrf2), two factors strictly related to the well-functioning of cellular antioxidant machinery [59], has also been frequently observed in PCa [60]. In addition to the above, it has been found that androgens are able to induce oxidative stress in both non-cancerous and PCa cells through the interaction with androgen receptor [61,62].

## 6. Immunogenic Basis of PCa

The immunogenic landscape of the PCa microenvironment is still not completely understood. The immune system can affect PCa through cellular infiltration or secretion of immune modulatory substances. Interferon-1 (INF-1) is essential for establishing an effective anti-tumour immune response, which can be obtained through a number of mechanisms such as cytokines production (e.g., tumour necrosis factor) [63]. However, its role in PCa is still not clear. INF-1 signalling is affected by the activity of the transcription factors signal transducer and activator of transcription 1 (STAT-1) and STAT-3. Cancer cells are made resistant to radiation and chemotherapy by a sub-population of globulins activated by unphosphorylated STAT-1 following sustained IFN-1 exposure [64]. When mice with only phosphatase and tensin homolog (PTEN) gene deficiency were compared to prostate-specific STAT-3- and PTEN-deficient animals, the latter showed accelerated cancer development and metastasis [65]. The conflicting results regarding the function of IFN-1 could be due to the variations in signal length and STAT activation.

In addition to the role played by cytokines, immune cellular infiltration in PCa has been investigated. The numerosity of tumour-infiltrating lymphocytes is functionally important and correlates with the clinical outcome observed in several types of tumours. CD3^+^ T cells have been associated with lower biochemical recurrence survival, while inflammatory lesions displayed more CD4^+^ T cells compared to CD8^+^ T cells, which are more prominent in normal prostatic tissue [66]. It has also been shown that intralesional infiltration with mast cells is associated with better prognosis and less aggressive behaviour of PCa [67]. However, other studies have shown longer progression-free survival with minimal infiltration with mast cells [68]. These controversial results could be related to the variety of cytokines produced by mast cells which might have different impact on PCa [69]. Compared to T cells and mast cells, limited data are available regarding the role of B-lymphocytes in PCa. With this regard, a study reported that B-lymphocytes activate STAT-3, which is an enhancement of the progression of CRPC [70].

In the current era of ICIs, PCa represents one of the cancer types that have been investigated with these novel drugs targeting PD-1, PD-L1, and CTLA-4 receptors [71]. It has been reported that mCRPC expresses a low level of PD-L1 receptors, which can be a limiting factor for significant response to ICIs. PD-L1 expression level might predict the response of mCRPC to enzalutamide, a second-generation anti-androgen medication [72]. Despite different studies on this topic, the predictive role of PD-L1 expression for response to IC is still under debate, especially in view of the heterogeneity of the results obtained considering different types of cancers [73]. Ipilimumab, an anti-CTLA-4, has been also investigated in mCRPC, showing a reduction of prostate-specific antigen (PSA) levels by more than 50% without significant side effects [74]. In addition, it displayed better clinical response when combined to ADT or radiotherapy, compared to the monotherapy [75].

## 7. Main Genes Involved in PCa

Patients with a family history of PCa represent about 9% of all PCa patients. Some families might have a higher risk of developing PCa due to genetic causes. The risk of developing PCa can vary from 2 to 11 times higher than normal, depending on the number of first-degree relatives diagnosed with PCa [76]. Understanding the genetic bases of PCa is fundamental for both identifying people with a higher risk and for expecting the behaviour of the disease in already diagnosed patients. A few dozen multiple genome-wide association studies (GWAS) identified more than 170 genetic variants. In this review, we highlighted the main genes involved in familial PCa [77] (Table 1).

### 7.1. BRCA1 and BRCA2

Both BRCA1 and BARCA2 are tumour suppressor genes located on chromosome 17q12–21 and 13q12–13, respectively. Around 3% and 10% of breast and ovarian cancer patients, respectively, present heredity mutation of these genes [78]. For people presenting a BRCA1 mutation, the Breast Cancer Linkage Consortium (BCLC) reported an increase in PCa risk in men aged <65 years, whereas no risk increase was observed for men aged ≥65 years [79]. According to the InforMing the Pathway of Chronic Obstructive Pulmonary Disease Treatment (IMPACT) trial, the BRCA2 gene mutation carries a higher risk of developing early onset PCa and more aggressive disease [80]. Furthermore, first-degree male relatives of women having breast or ovarian cancer are at higher risk of developing PCa [81]. Patients with BRCA2 mutation might show less response to taxane-based chemotherapy [82].

### 7.2. HOXB13

The gene HOXB13 encodes a transcription factor that belongs to the homeobox gene family. It normally acts as a tumour suppressor gene to protect from cancer [83]. G84E mutation of HOXB13 gene has been found in about 3% of familial and early onset PCa. Patients who carry this HOXB13 mutation are at higher risk of disease recurrence after definitive treatment [84]. Additionally, germline HOXB13 G84E mutation has been associated with other cancers such as rectosigmoid and non-melanoma skin cancers, as shown in a recent study considering subjects from the U.K. Biobank [85].

### 7.3. NKX3.1

NKX3.1 is a transcription factor protein composed of 234 amino acids expressed in the prostate. It is a PSA-regulated homeobox gene, located on chromosome 8p21. Numerous primary prostatic adenocarcinomas show positive staining for NKX3.1 protein, while it is completely lost in about 75% of metastatic disease [86]. This protein displayed almost 100% sensitivity and specificity as in vitro biomarker for metastatic prostatic carcinoma. It has been utilised as a diagnostic marker for PCa and other metastatic diseases originating in the prostate [87].

### 7.4. MYC

The MYC family represents a group of three different proto-oncogenes, namely, c-myc (MYC), l-myc (MYCL), and n-myc (MYCN). Since c-myc was the first gene discovered, it is usually identified as MYC [88]. Mutations of MYC were observed in very early stages of PCa as well as in PIN. On the basis of animal studies, MYC mutation could be responsible of initiation of PIN, followed by progression to adenocarcinoma [89]. Pre-clinical studies suggested that MYC-targeted therapy might be a novel approach for the treatment of CRPC [90,91].

### 7.5. PTEN

PTEN is a classical tumour suppressor gene located in the 10q23 region of chromosome 10. Deletion and/or mutation of PTEN was detected in about 40% of PCa, correlating with more aggressive forms of the disease [92]. Its clinical applications are still under investigation; however, it could be used as a prognostic marker to help in triaging patients undergoing active surveillance or radical treatment. Furthermore, patients with PTEN loss could be more responsive to ICIs [93].

### 7.6. TMPRSS2–ERG Fusion

TMPRSS2 is a cell surface protein encoded by a gene located on chromosome 21 and mainly expressed by endothelial cells part of the respiratory and digestive tracts [94]. Until now, the exact biological function of TMPRSS2 is unclear. ERG is an oncogene, located on chromosome 21, that plays a key regulatory role of cell proliferation, differentiation, angiogenesis, inflammation, and apoptosis [95]. TMPRSS2–ERG fusion was found in about 50% of PCa cases and associated with the upregulation of ERG gene, more aggressive disease, and higher mortality [96]. Preclinical studies suggested that TMPRSS2–ERG fusion could have a regulatory role on androgen receptors pathway, also reducing the responsiveness of PCa to new antiandrogens such as enzalutamide [97].

### 7.7. Forkhead Box A1 (FOXA1)

FOXA1 gene encodes for forkhead box protein A1, also known as hepatocyte nuclear factor 3-alpha (HNF-3A). Some studies have shown a particular role for FOXA1 in the postnatal development of the prostate [98]. Furthermore, FOXA1 is able to influence androgen receptor (AR) signalling through direct interaction, regulating the development and survival of normal prostate and PCa cells [99]. FOXA1 also regulates the epithelial-to-mesenchymal transition (EMT) in an AR-independent manner [100]. It has been shown that mutations in the coding sequence and *cis*-regulatory elements of FOXA1 cause functional changes in PCa [101]. A recent study demonstrated that the inhibition of the associated cofactor LSD1 changes the methylation status of FOXA1, resulting in chromatin dissociation and tumour suppression, even in treatment-resistant PCa [102].

## 8. Mechanism of Resistance to ADT

ADT is a main treatment component for advanced and metastatic PCa and is intended to either prevent testosterone production or to directly prevent it from acting on PCa cells [103]. About 20% of patients might develop resistance to ADT within a few years of starting the treatment [104]. Even with very low testosterone level and despite the administration of the novel second-generation antiandrogens, some patients can still encounter the progression of the disease. Therefore, complete understanding of ADT resistance by PCa represents a very dynamic area for researchers [105]. PCa growth and progression are driven mainly through stimulation of AR signalling; indeed recent studies have suggested that, despite the significant reduction in testosterone level, AR signalling is still involved in disease progression. Therefore, blocking of this pathway is the main aim for most of the new therapeutic agents acting against advanced and metastatic PCa [106].

**Figure 1:**
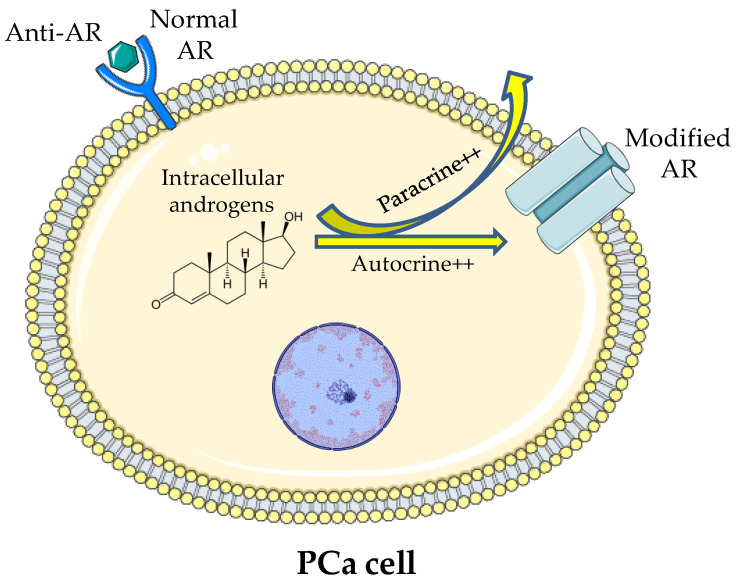
Illustration of the theory of intracellular production of androgen and stimulation of modified ARs occurring despite the low serum testosterone and the blockage of normal ARs.

The resistance to the treatments showed by PCa could be due to an adaptive mechanism of microenvironment. In addition, PCa cells might be able to produce androgens and modify the AR, which allows the maintenance of the signalling even in the presence of low serum testosterone [107] (Figure 1).

In addition to the previous theories, other genetic abnormalities could explain the progression of the tumour despite AR blockage; to name a few, the AR gene mutation and/or overexpression, the expression of AR splicing variants, and the upregulation of transcriptional co-activators [108]. In a study carried out by Korpal et al., it has been demonstrated as the F876L mutation in AR confers genetic and phenotypic resistance to MDV3100 (enzalutamide) in LNCaP androgen-sensitive human prostate adenocarcinoma cells. In particular, F876L mutation in AR was associated with a reduced AR response to this drug and sustained cell proliferation despite the therapy [109]. Studies employing CRPC xenografts have shown that several genes involved in the androgen synthesis pathway, including CYP17A1, are over-expressed during hormonal therapy [110]. It has also been demonstrated that AR mutations can be found in up to 30% of CRPC patients under ADT; interestingly, the treatment with new antiandrogens could enhance their incidence favouring the clonal selection of tumour cells through the suppression of AR signalling, also increasing AR somatic mutations and the consequent abnormal transcription [111].

## 9. Conclusions

Development and progression of PCa have been deeply explored but not completely understood. This tumour involves numerous inflammatory, immunological, and genetic pathways that significantly affect the directions of targeted therapy. On the basis of the current understanding of the natural behaviour of PCa, the use of patients’ genetic profiling might help to optimise the administration of a personalised and effective therapy, also predicting the patients’ response before starting the treatment.

## Figures and Tables

**Table 1:** Summary of the main genes involved in prostate cancer.

Gene	Location	Percentage	Normal Function	Abnormality
BRCA2	13q12	13%	Tumour suppressor	Mutation
HOXB13	17q21-22	3%	Tumour suppressor	G84E mutation
NKX3.1	8p21	75%	Negative regulator of epithelial cell growth in prostate	Downregulation to complete loss
MYC	2p24	50%	Multiple functions including cell cycle, cell growth, and apoptosis regulation	Overexpression
PTEN	10q23	40%	Tumour suppressor	Loss
ERG	21q22	50%	Transcriptional regulator	Fusion with TMPRSS2
FOXA1	14q21	41%	Transcriptional regulator	Mutation

## Data Availability

Not applicable.
